# Weight Gain and Tenderness in Nelore Cattle: Genetic Association and a Potential Pleiotropic Role of Transcription Factors and Genes

**DOI:** 10.3390/ani15192874

**Published:** 2025-09-30

**Authors:** Elora R. P. de S. Borges, Lucio F. M. Mota, Lucas L. Verardo, Lucia G. de Albuquerque, Marcela R. Duarte, Geovana C. Santos, Alice S. Pereira, Lorena M. P. de Carvalho, Lilia S. Carvalho, Emily A. R. Almeida, Ana F. B. Magalhães

**Affiliations:** 1 Laboratory of Animal Breeding, Department of Animal Science, Universidade Federal dos Vales do Jequitinhonha e Mucuri, Rodovia MGT 367, Km 583, n° 5000, Alto da Jacuba, Diamantina 39100-000, MG, Brazil; elora.borges@ufvjm.edu.br (E.R.P.d.S.B.); lucas.verardo@ufvjm.edu.br (L.L.V.); marcela.ramos@ufvjm.edu.br (M.R.D.); geovana.santos@ufvjm.edu.br (G.C.S.); alice.pereira@ufvjm.edu.br (A.S.P.); lorena.carvalho@ufvjm.edu.br (L.M.P.d.C.); lilia.carvalho@ufvjm.edu.br (L.S.C.); emily.alves@ufvjm.edu.br (E.A.R.A.); 2 Department of Animal Science, School of Agricultural and Veterinary Sciences, São Paulo State University (UNESP), Jaboticabal 14884-900, SP, Brazil; flaviommota.zoo@gmail.com (L.F.M.M.); galvao.albuquerque@unesp.br (L.G.d.A.)

**Keywords:** beef cattle, genetic parameters, genomics, GWAS, meat quality

## Abstract

The inclusion of meat quality traits in breeding programs is a promising strategy to improve beef by selecting animals based on both growth and meat quality. This study aimed to estimate genetic parameters for average daily gain (ADG) and Warner–Bratzler shear force (WBSF), as well as to perform genome-wide association studies (GWAS) to identify genomic regions and transcription factor (TF) binding sites associated with both traits in Nelore cattle. Genetic parameters were estimated using a bi-trait Bayesian model, and GWAS identified key SNPs explaining over 1% of variance in genomic estimated breeding values. Candidate genes near these SNPs were annotated, TF binding sites predicted, and gene–TF networks constructed. Genetic estimates indicated moderate heritability for ADG, low heritability for WBSF, and a small negative genetic correlation between traits. Genomic regions contained 116 and 151 candidate genes for ADG and WBSF, respectively, with 35 shared between traits. Functional analyses highlighted *MYBPC1* and *PENK* for WBSF, and *GHRS* and *NPY* for ADG. TF analysis identified 25 TFs, with 3 key ones highlighted. Gene–TF networks revealed candidates including *CAPN1* and *LTBP3* for WBSF, and *CARM1* and *GH1* for ADG. Shared candidate genes identified in the combined network provide valuable insights into the genetic architecture of growth and tenderness in Nelore cattle.

## Simple Summary

Beef quality is important for both farmers and consumers. Traditionally, cattle are selected based on weight gain, as faster-growing animals are considered more profitable. However, faster growth does not always mean the meat will be tender. This study investigated the genetic factors influencing growth and meat tenderness in Nelore cattle. We analyzed phenotypic and genotypic data from young bulls to understand the genetic relationship between weight gain and tenderness and to identify specific regions in the DNA associated with these traits. Some genes influenced weight gain and meat tenderness, while others were related to only one trait. We also examined how these genes are regulated and interact, identifying key genetic elements that could help breeders select animals with better overall performance. Understanding these genetic relationships allows farmers to make informed breeding decisions to produce animals that grow efficiently and provide high-quality meat. These findings are valuable for improving beef quality, benefiting producers, gaining more efficient and profitable animals, and consumers who prefer tender and high-quality meat.

## 1. Introduction

Brazil is the largest beef exporter in the world, accounting for a fourth of all beef exports globally [1]. It is the second-largest beef producer [1], with the Nelore breed (*Bos indicus*) and its crossbreeds representing around 80% of the Brazilian herd [2], as they adapt to different regions of the country. However, historically, zebu meat was considered tough, as these animals were raised extensively and took longer to slaughter compared to early breeds of European cattle [3]. In addition, the meat exported by Brazil is considered of medium and low quality [4] by international buyers, as the meat tenderness represents a significant challenge in the Zebu beef cattle industry, not reaching the revenue equivalent to production capacity.

One of the most important selection criteria is the average daily weight gain, as it represents an indicator of growth rate, which measures how much weight an animal gains in each period and directly impacts the productivity of cattle breeding systems. However, traits associated with the meat quality of this breed have been forgotten for a long time, since they are difficult to measure and require the slaughter of animals. Therefore, most Nellore breeding programs have historically focused on improving reproductive and weight gain traits, which may be a determining factor in this scenario.

Meat tenderness represents a crucial attribute that influences consumer satisfaction and market value, directly impacting the profitability of beef cattle farming. This trait can be estimated using the WBSF (Warner–Bratzler shear force) procedure. However, it is difficult to measure, since it requires specific laboratory equipment, and it is measured after the slaughter of animals and can be expensive [5,6].

A tool that has been used to improve traits that are difficult to measure is genomics, which improves the precision of estimated genetic values and facilitates the prediction of traits that are difficult to assess [7], as in the case of tenderness. According to [8], using genomic information in animal selection reduces the generation intervals and can reduce costs by up to 90% compared to traditional genetic improvement programs. Therefore, incorporating genomic information into genetic evaluations may provide a promising approach for obtaining more accurate estimates of genetic parameters [9], particularly for complex or costly phenotypes. Nevertheless, research on the relationship between weight gain and meat tenderness in the Nelore breed has been restricted to correlation estimates based on pedigree and phenotypic data obtained using traditional methods [10].

Genotypic data can also be used in genome-wide association studies (GWAS) to identify and map genomic regions associated with economically important traits. Given the economic relevance of growth and meat tenderness in beef cattle, GWAS has been applied to detect loci linked to these traits [5,11,12]. However, most of these studies emphasize the biological interpretation of the identified candidate genes, while the functional relationships with transcription factors (TFs) remain unexplored. Transcription factors are key regulators of gene expression [13] and have been investigated in different livestock species [13,14,15,16,17,18]. Therefore, we argue that incorporating TF analyses may improve understanding of the mechanisms underlying complex traits, offering a more effective framework for detecting pleiotropic effects and uncovering shared genetic architecture.

This paper aimed to estimate the genetic parameters for weight gain and meat tenderness of Nelore cattle using genotypic data and to identify the genomic regions that play a role in these traits’ biological processes and transcription factor binding sites.

## 2. Material and Methods

### 2.1. Sample Collection

The data set was obtained from the records of 6277 young Nelore bulls, including phenotypic information for ADG measured from weaning (221 ± 33.51 days old) to the year (523 ± 50.70 days old). Of these, 5555 young bulls had phenotypic data for WBSF (Warner–Bratzler shear force) of animals slaughtered at 703.9 ± 79.69 days old. The animals with phenotypic data of WBSF were only intact males, born between 2008 and 2010, raised in grazing systems, with some farms offering protein and mineral supplementation during the dry season, while others offer only urea and mineral supplementation, and feedlot finished. During the feedlot period, the animals were fed with a total mixed ration (dry corn grain, corn silage, sugarcane bagasse, soybean, urea, mineral salt, and potassium chloride) and slaughtered in commercial slaughterhouses. These animals were part of four different breeding programs: DeltaGen, Cia do Melhoramento, and Paint breeding programs that integrate the Alliance Nelore database (www.gensys.com.br, accessed 4 August 2025) and Nelore Qualitas (www.qualitas.agr.br, accessed 4 August 2025) breeding program.

Meat samples were collected in commercial slaughterhouses in Brazil’s different states: São Paulo, Mato Grosso, Mato Grosso do Sul, Goiás, and Bahia. These slaughterhouses were registered by the Federal Inspection Service (SIF) of the Ministry of Agriculture, Livestock and Supply (MAPA). Thus, according to legislation, the carcasses were cooled until they reached a temperature equal to or lower than 5 °C, within 24 and 48 h post-mortem. These samples were collected from the *Longissimus thoracis* muscle at the interface of the 12th and 13th ribs, taken from the left half of the carcass, frozen, and transported to the laboratory. All samples remained frozen, and no aging process was applied. Animals from the same farm and birth year were slaughtered at the same slaughterhouse on the same date, and their meat was analyzed on the same day using the Warner–Bratzler shear force (WBSF) method. Further details regarding meat analysis can be found in [5,12].

We defined contemporary groups (CGs) for the ADG and WBSF groups based on year, season of birth, herd (at birth, weaning, and yearling stages), and slaughter date for WBSF. We split the birth season into two periods: August to January and February to July. To ensure data quality, we excluded observations with measurements of more than 3.5 standard deviations above or below the CG mean, and CGs with fewer than five animals. Table 1 shows the descriptive statistics for each trait after data quality control.

A total of 20,859 genotyped Nelore animals were included in this research. These animals were genotyped using various Bead chip assay densities (Supplementary Materials—Table S1). Animals with lower-density genotyping panels were imputed to the high-density (HD) panel using FImpute v3 software [19]. Before imputation, markers situated in non-autosomal regions or having the exact genomic coordinates were removed, followed by a quality control (QC) filter to remove autosomal SNPs with a GenCall score <0.6 to remove genotyping problems [20,21].

Low- to moderate-density SNP genotypes were imputed to the HD panel using a reference panel of 6862 animals, comprising 3107 included in the present study and 3755 genetically linked to the target population. The genomic QC for imputed animals was performed using the qcf90 programs [22], removing genetic markers: (a) with a call rate < 0.90, (b) with a minor allele frequency (MAF) < 0.05, (c) with deviation from HWE < 0.15, and (d) monomorphic markers. In addition, samples with a call rate < 0.90 and Mendelian conflict were also removed. After QC, 20,745 animals and 377,248 SNP markers remained for further analysis. All details about the genotyping process were described by Arikawa et al. [2].

### 2.2. Statistical Analysis

#### 2.2.1. Estimation of (co)Variance Components

The genetic (co)variance components for ADG and WBSF were obtained with a bi-trait animal model using the Bayesian approach:

$$\left[\begin{array}{c} y_{1}\\ y_{2} \end{array}\right]=\left[\begin{array}{cc} X_{1} & 0\\ 0 & X_{2} \end{array}\right]\left[\begin{array}{c} b_{1}\\ b_{2} \end{array}\right]+\left[\begin{array}{cc} Z_{1} & 0\\ 0 & Z_{2} \end{array}\right]\left[\begin{array}{c} a_{1}\\ a_{2} \end{array}\right]+\left[\begin{array}{c} e_{1}\\ e_{2} \end{array}\right]$$

where $y_{1}$—records vector of the WBSF trait; $y_{2}$—records vector of the ADG trait; $b_{1}-$ vector of fixed effects for the WBSF trait (contemporary group); $b_{2}$—vector of fixed effects for the ADG trait (group of contemporaries); $a_{1}$—vector of random effects of genetic value for the WBSF trait; $a_{2}$—vector of random effects of genetic value for the ADG trait. $X_{1}$ and $X_{2}$ represents the incidence matrices associating the fixed effects $b_{1}$ and $b_{2}$ to phenotypes $y_{1}$ and $y_{2}$, respectively; and $Z_{1}$ and $Z_{2}$ are the incidence matrix relating $y_{1}$ and $y_{2}$ to the additive genomic breeding value $a_{1}$ and $a_{2}$.

The structure of variances and covariances of the random effects of the bi-trait animal model is

$$Var\left[\begin{array}{c} a_{1}\\ a_{2}\\ \begin{array}{c} e_{1}\\ e_{2} \end{array} \end{array}\right]=\left[\begin{array}{c} \begin{array}{cc} {A\sigma }_{a_{1}}^{2} & \begin{array}{cc} {A\sigma }_{a_{1,2}} & \begin{array}{cc} 0 & 0 \end{array} \end{array} \end{array}\\ \begin{array}{cc} {A\sigma }_{a_{1,2}} & \begin{array}{cc} {A\sigma }_{a_{2}}^{2} & \begin{array}{cc} 0 & 0 \end{array} \end{array} \end{array}\\ \begin{array}{c} \begin{array}{cc} \;\;\;0\;\; & \begin{array}{cc} \;\;\;\;\;\;0 & \begin{array}{cc} {I\sigma }_{e_{1}}^{2} & 0 \end{array} \end{array} \end{array}\\ \begin{array}{cc} \;\;\;0\; & \begin{array}{cc} \;\;\;\;\;\;\;0 & \begin{array}{cc} 0 & {I\sigma }_{e_{1}}^{2} \end{array} \end{array} \end{array} \end{array} \end{array}\right]$$

where $\sigma_{a_{1}}^{2}$ and $\sigma_{a_{2}}^{2}$ are the variances of the direct additive genetic effect; $\sigma_{e_{1}}^{2}$ and $\sigma_{e_{e}}^{2}$ are the residual variances for the WBSF and ADG traits, respectively; $\sigma_{a_{1,2}}$ is the additive genetic covariance between the traits.

The bivariate animal model was implemented using the Gibbsf90 + software from the BLUPf90 family [23]. The Gibbs sampler consisted of a chain of 500,000 cycles, with a burn-in of the first 50,000 iterations and samples stored every ten cycles. Therefore, the posterior means of genetic parameters were estimated from 45,000 samples, with convergence assessed through visual inspection of the trace plot using the BOA package (version 1.1.8-2) in R (version 4.3.0) [24], and all traits showed convergence (*p*-value > 0.15) according to the Geweke test [25].

#### 2.2.2. GWAS Analysis

The GWAS analysis was performed considering the multivariate elastic net (ENET) penalized regression model, which represents a robust approach to dealing with correlated QTLs (quantitative trait loci), which combines $l_{1}=\sum {\left\|W\right\|}_{2,1}$ (least absolute shrinkage and selection operator—LASSO) and $l_{2}=\;\sum {\left\|W\right\|}_{F}^{2}$ (ridge regression—RR) penalties to handle the high-dimensional SNP data and the strong correlations among markers in linkage disequilibrium (LD). The $l_{1}$ and $l_{2}$ penalty terms are controlled by the alpha parameter (α), providing a balance between selection (LASSO) and shrinkage (RR) of predictor variables (SNP markers). The optimum weight values for λ and α in the Enet are considered in the least square loss function as follows:

$$W\left(\lambda ,\alpha ,\beta \right)=\begin{array}{c} min\\ W \end{array}\left[\frac{1}{2N}{\left\|Y-XW\right\|}_{F}^{2}+\lambda \left(\alpha {\left\|W\right\|}_{2,1}+(1-\alpha ){\left\|W\right\|}_{F}^{2}\right)\right]$$

where ${\left\|W\right\|}_{2,1}=\sum_{j=1}^{p}{\left\|W_{j}\right\|}_{2}$ enforces row-sparsity (shared SNP selection across traits; pleiotropy), and ${\left\|W\right\|}_{F}^{2}$ stabilizes estimates under LD. Hyperparameters (λ, α) were tuned by random search, in which λ controls the strength. This formulation follows the multi-task feature-learning implemented using the glmnet R package (version 4.1-7) [26], considering a Gaussian family.The ENET penalties obtained during the GWAS analysis were used in a forward validation scheme, aiming to evaluate the predictive ability of the ADG and WBSF traits. The random search for α and λ parameters was performed using the caret R package (version 6.0-94) [27]. We divided the data set based on birth year, where the training set covers animals born from 2008 to 2016 (*n* = 4402), and the validation set includes animals from 2017 to 2018 (*n* = 376). During the learning process of the ENET approach, the training set was split into an 80:20 ratio, and the trained model with the highest accuracy and lowest mean square error (MSE) was then applied to a separate validation set. The predictive ability of the ENET model was 0.91 for ADG and 0.88 for WBSF.

SNP markers with a relative importance score explaining more than 1% of the variability in GEBV (Genomic Estimated Breeding Value) for ADG and WBSF were considered significant. SNP importance was quantified as the fraction of predictive variance explained in the validation set, computed as the decrease in predictive ability (R^2^) when the SNP was permuted while keeping all other predictors and coefficients fixed [28]. SNPs with importance score > 1% were considered significant, based on three criteria: (i) this threshold corresponds to a biologically interpretable contribution (partial R^2^ > 0.01); (ii) it lies above the null distribution, as no SNP exceeded 0.5% in 1000 phenotype permutations (95th percentile); and (iii) it provided the best compromise between sensitivity and robustness in bootstrap and cross-validation analyses.

Significant SNP markers identified by the ENET analysis were clustered into chromosomal regions. We defined windows of ±100 kb around each significant SNP and annotated genes located within these intervals for gene identification. Candidate genes were identified using the NCBI Genome Data Viewer (https://www.ncbi.nlm.nih.gov/genome/gdv/, accessed on 29 September 2025), which allowed inspection of the bovine reference genome (ARS-UCD1.2) and retrieval of gene models overlapping or proximal to the detected regions.

#### 2.2.3. Pós-GWAS Analysis

Gene networks highlighting biological processes related to candidate genes of ADG and WBSF traits were constructed using the ClueGO application, using the bovine gene data set as a background, which visualizes non-redundant biological terms for large gene clusters in a functionally clustered network. This application is part of Cytoscape v3.10.0 software, an open-source bioinformatics platform for visualizing molecular interaction networks and integrating them with gene expression profiles and other contextual data. This analysis was performed based on bilateral hypergeometric tests and the Bonferroni correction, using the ClueGo default parameters (medium network specificity and Kappa Score = 0.4) with no *p*-value cutoff, aiming to explore the maximum information of the biological process from the bovine data set.

To search for transcription factor binding sites (TFBS), the TFMExplorer programme (http://bioinfo.lifl.fr/TFM/TFME; accessed on 29 September 2025) was utilized, which employs weight matrices from the JASPAR vertebrate database [29] to detect potential TFBS, calculating a scoring function as described in [30]. We selected sequences of 3000 bp upstream and 300 bp downstream (FASTA format) from the transcription start site, based on the ARS-UCD1.3 reference genome, available at NCBI databases (https://www.ncbi.nlm.nih.gov/; accessed on 29 September 2025), and used as input for TFM-Explorer with default parameters (Number of clusters to display = 25; maximum *p*-value of 0.001; ratio density of 2.5). The obtained list of TFs was analyzed in Cytoscape [31] via the BinGO plug-in [32] to determine significant gene ontology terms, assuming standard analyses and multiple corrections test (*p*-value <  0.05).

We utilized the Network Analyzer tool in Cytoscape v3.10.0 to highlight the most relevant candidate genes based on the number of TF–gene network connections. These analyses enriched genes and TF based on the number of TFBS and connections in each gene and TF link, determining the most connected genes in the TF–gene network.

## 3. Results

### 3.1. Estimation of Genetic Parameters

The estimation of genetic parameters (Table 1), using genotypic data, exhibited low magnitude values for WBSF and a moderate estimate for ADG. Meanwhile, the genetic correlation between WBSF and ADG was negative, although it was of low magnitude.

### 3.2. GWAS

**Figure 1:**
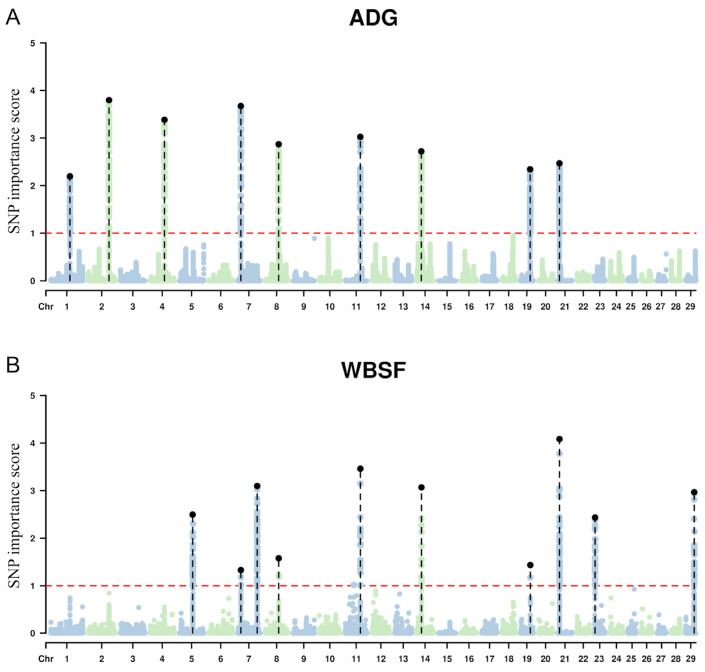
Manhattan plots for (**A**) average daily gain (ADG) and (**B**) Warner–Bratzler shear force (WBSF) in Nelore cattle. The *Y*-axis shows each SNP marker’s relative importance score (RIS) of each SNP marker estimated from elastic net penalized regression, reflecting the normalized magnitude and selection stability of SNP effects. The dashed red line indicates the empirical threshold (RIS ≥ 1.0) used to highlight influential SNPs. The *x*-axis shows genomic positions based on the ARS-UCD1.2 bovine reference genome.

The genomic regions that explained more than 1% of the additive genetic variance, considering the relative importance scores calculated for each SNP in elastic network analyses, were mapped on BTA 1, 2, 4, 7, 8 11, 14, 19, and 21 for ADG; BTA 5, 7, 8, 11, 14, 19, 21, 23 and 29 for WBSF (Figure 1).

The genomic regions containing the location (chromosome and position), SNP numbers, size (Mb), and importance score harbored 116 and 151 candidate genes for ADG and WBSF, respectively (Table 2 and Table 3). In addition, we found 35 candidate genes in common to both traits: *DOCK6*, *RAB3D*, *TMEM205*, *CCDC159*, *PLPPR2*, *SWSAP1*, *EPOR*, *RGL3*, *ODAD3*, *PRKCSH*, *ELAVL3*, *ZNF653*, *ECSIT*, *LOC508834*, *INSR*, *ARHGEF18*, *PEX11G*, *LPL*, *DTNB*, *DNMT3A*, *MIR1301*, *POMC*, *EFR3B*, *TMEM68*, *TGS1*, *LYN*, *RPS20*, *LOC112449628*, *LOC112449630*, *MOS*, *PLAG1*, *LRRC28*, *TTC23*, *SYNM*, and *IGF1R*.

### 3.3. Pós-GWAS

**Figure 2:**
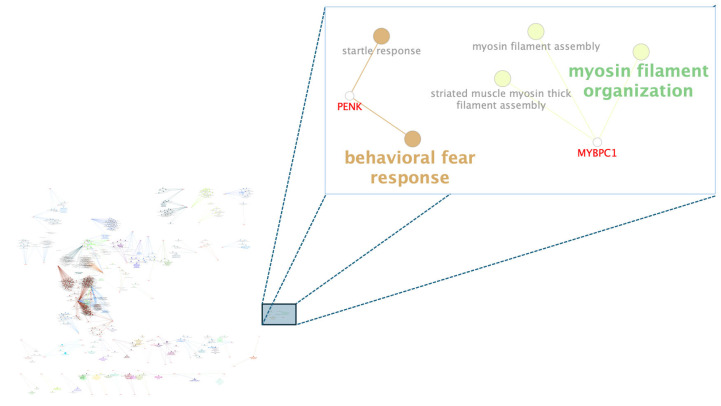
Functional networks between genes and biological processes associated with Warner–Bratzler shear force (WBSF). Main interaction networks between biological processes and genes (nodes) are shown in a zoom. The size of the node characterizes the enrichment of the process according to ClueGO Cytoscape plug-in [33]. The terms that are most enriched by subnet are in bold.

**Figure 3:**
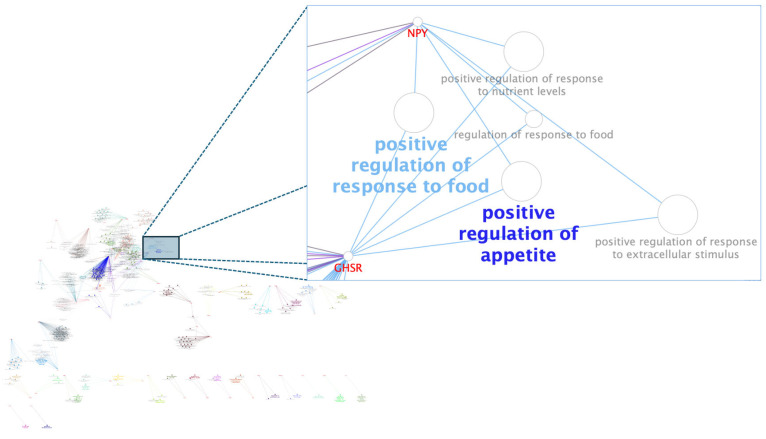
Functional networks between genes and biological processes associated with average daily gain (ADG). Main interaction networks between biological processes and genes (nodes) are shown in a zoom. The size of the node characterizes the enrichment of the process according to ClueGO Cytoscape plug-in [33]. The terms that are most enriched by subnet are bold.

Among the biological processes found (Figure 2 and Figure 3), the *MYBPC1* and *PENK* genes stood out for participating in reactions directly related to WBSF. In contrast, in the average daily weight gain of the population, the candidate genes that stood out were *GHRS* and *NPY*.

### 3.4. Gene-Transcription Factor

**Figure 4:**
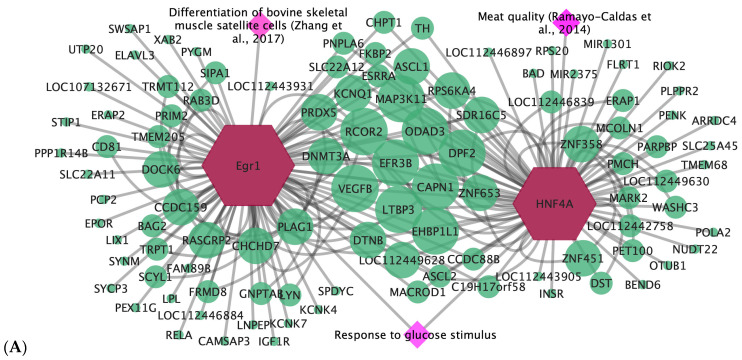

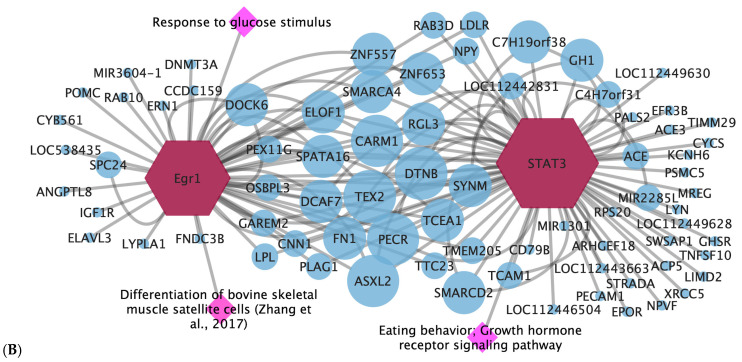
Gene-transcription factors network for Warner–Bratzler shear force (WBSF) and average daily gain (ADG) in Nelore cattle. (**A**) Transcription factors (red hexagon nodes) associated with candidate genes (circle nodes in green) for WBSF. Pink diamond nodes represent the biological processes and literature evidence associated with transcription factors. The size of the nodes represents the enrichment as a function of the number of transcription factor binding sites (TFBS); as the number of TFBS increases, the size of the nodes increases. (**B**) Transcription factors (red hexagon nodes) associated with candidate genes (circle nodes in blue) for ADG. Pink diamond nodes represent the biological processes and literature evidence associated with transcription factors. The size of the nodes represents the enrichment as a function of the number of transcription factor binding sites (TFBS); as the number of TFBS increases, the size of the nodes increases. Literature cited are Zhang et al., 2017 Clique ou toque aqui para inserir o texto [35] and Ramayo-Caldas et al., 2014 [36].

**Figure 5:**
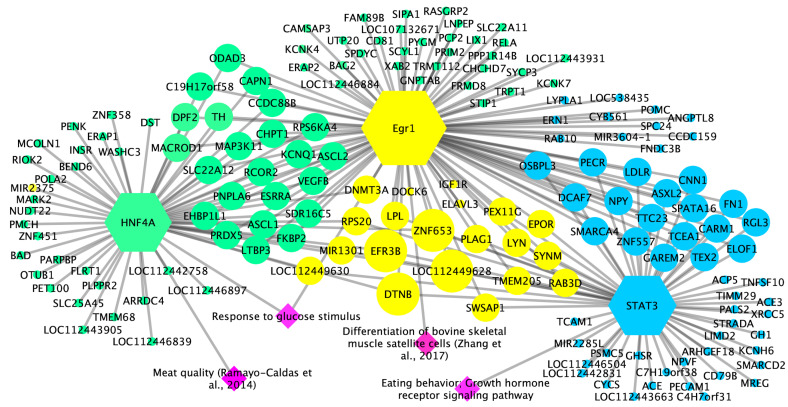
Combined gene-transcription factors network for Warner–Bratzler shear force (WBSF) and average daily gain (ADG) in Nelore cattle. Transcription factors (hexagon nodes) associated with candidate genes (circle nodes) for WBSF (green) and ADG (blue). Common transcription factors and genes are in yellow. Pink diamond nodes represent the biological processes and literature evidence associated with transcription factors. The size of the nodes represents the enrichment as a function of the number of transcription factor binding sites (TFBS); as the number of TFBS increases, the size of the nodes increases. Literature cited are Zhang et al., 2017 [35] and Ramayo-Caldas et al., 2014 [36].

According to TFM-Explorer program, 25 TF were related to the candidate genes for WBSF and ADG (Supplementary Materials—Table S2). Based on biological processes (Supplementary Materials—Table S3 for WBSF and ADG, respectively) that were associated with each trait according to BinGO Cytoscape plug-in [32] and literature evidence linking the TF with each studied trait, three key TF associated with ADG and WBSF in Nelore cattle were selected as presented in Table 4. These key TF were used to generate gene-TF networks for each trait (Figure 4), enabling the highlighting of promising candidate genes. Thus, from the 267 candidate genes observed, promising candidate genes for WBSF (e.g., *CAPN1* and *LTBP3*) and ADG (e.g., *CARM1* and *GH1*) in Nelore cattle were highlighted in this study. Moreover, considering that we observed common genes and TF, a combined gene-TF network was obtained, highlighting candidate genes for both ADG and WBSF (e.g., *LOC112449628*, *ZNF653*, *EFR3B*, *DTNB*, and *PLAG1*) (Figure 5).

## 4. Discussion

### 4.1. Estimation of Genetic Parameters

The moderate heritability estimate for ADG indicates that this trait can respond effectively to genetic selection. This outcome was anticipated, since such traits have long been emphasized in beef cattle breeding programs because of their economic relevance, relatively high heritability, and ease of recording. Growth-related traits have consistently been prioritized in selection schemes, with heritability estimates ranging from moderate [37,38] to high [10]. Nonetheless, most previous studies have based heritability estimates for weight gain exclusively on phenotypic and pedigree information, highlighting the still limited incorporation of genomic data as presented by this study.

The low heritability estimate for WBSF indicates limited potential for genetic progress through conventional selection, highlighting that environmental factors, such as pre-slaughter management and nutritional interventions, or non-additive genetic effects, play a major role in explaining most observed variation. Similar estimates for both traits, based on genomic information, were also reported by [39,40].

The estimated genetic correlation between weight gain and tenderness was negative, indicating that an increase in ADG tends to reduce shear force, which is favorable since lower shear force values correspond to greater meat tenderness. Nevertheless, this result should be interpreted with caution, as the magnitude of the genetic correlation was low. A low, although positive, correlation was reported by [10] and studies estimating correlations between weight gain and meat quality traits using genomic data remain scarce in the literature.

### 4.2. Pós-GWAS

The *MYBPC1* gene, found as a candidate gene for WSBF, encodes an isoform of myosin-binding protein C that plays a crucial role by interacting directly with the thin and thick filaments in the sarcomere of muscle fibers [41]. Studies related to the cardiac isoform of *MYBPC1* highlight its direct binding to actin and myosin filaments, which may contribute to the assembly and stabilization of the actomyosin complex [42,43]. Moreover, this gene was cited as a strong biomarker of postmortem meat quality in lamb [44]. Also identified as a candidate gene for WSBF, the *PENK* gene was associated with fear response in the gene-biological process network. A review carried out by [45] identified that fear, mainly when caused by failures in humane handling or inadequate treatment during transport, reception, and driving in slaughterhouses, is one of the leading causes of decreased glycogen storage and, consequently, a reduction in the quality of the meat and its tenderness. Moreover, this gene was identified in homozygous regions of the Brahman cattle breed [14], which is reported to be considered a more reactive breed to humans when compared with taurine breeds, such as Angus and Hereford [46]. 

Considering the ADG trait highlighted candidate genes, the *GHSR* gene is linked to the biological processes that regulate animals’ appetite and response to food intake, as is the *NPY* gene. Neuropeptide Y (*NPY*) is known as the most potent appetite regulator in terms of physiological function. Furthermore, it plays a role in regulating energy homeostasis, influencing reproductive function, and acts as one of the intermediaries of leptin, a protein produced by fat cells [47,48]. Based on these physiological and biological functions, *NPY* was identified as a candidate gene associated with cattle carcass traits and meat quality, such as feed intake, body fat deposition, and obesity, as highlighted by [48].

In this study, 35 candidate genes affecting the two studied traits were found, which were *DOCK6*, *RAB3D*, *TMEM205*, *CCDC159*, *PLPPR2*, *SWSAP1*, *EPOR*, *RGL3*, *ODAD3*, *PRKCSH*, *ELAVL3*, *ZNF653*, *ECSIT*, *LOC508834*, *INSR*, *ARHGEF18*, *PEX11G*, *LPL*, *DTNB*, *DNMT3A*, *MIR1301*, *POMC*, *EFR3B*, *TMEM68*, *TGS1*, *LYN*, *RPS20*, *LOC112449628*, *LOC112449630*, *MOS*, *PLAG1*, *LRRC28*, *TTC23*, *SYNM*, and *IGF1R*. Gene-biological process networks demonstrated that some genes can potentially control the manifestation of ADG and WBSF. Furthermore, among these, the PLAG1 and TMEM68 genes were also found by [5], who studied GWAS for tenderness and marbling. The authors highlighted that these genes are related to growth and muscle development in cattle.

In this study, based on candidate genes for WBSF and ADG, we identified three key transcription factors (STAT3, EGR1, and HNF4A) according to biological processes related to the studied traits (e.g., eating behavior, growth hormone receptor signaling pathway, response to hormone stimulus). STAT3 was identified as a TF for ADG in this study and has been cited to be associated with growth hormone secretion [34]. In addition, identified in both ADG and WBSF candidate genes, there is evidence that EGR1 promotes the differentiation of muscle satellite cells [35] and, thus, may affect meat traits and muscle development. The third TF, HNF4A, is pointed out as one of the regulators of carcass intramuscular fat deposition in beef cattle [36] and thus may impact meat tenderness. This way, these TF were pointed out here as key TF for ADG and WBSF and were used to highlight candidate genes for each trait in Nelore cattle.

Among the candidate genes for ADG, the *CARM1* and *GH1* genes are evident in our gene-TF network. The *CARM1* gene encodes the coactivator-associated arginine methyltransferase 1 protein and has been cited to play a role in the determination, maintenance, and plasticity of muscle in mice [49]. Moreover, the *GH1* encodes for the growth hormone 1 protein, with several studies associating it with growth and carcass traits in cattle [50,51,52].

Considering the candidate genes for WBSF, we observed the *LTBP3* and *CAPN1* genes with binding sites for the two key TF in the network. The LTBP3 encodes the latent transforming growth factor beta binding protein 3 and may also be a potential candidate gene for meat tenderness in pigs [53]. In addition, the *CAPN1* gene encodes calpain 1 protein, which has been associated with meat tenderness in cattle [52,54].

Moreover, it is important to note that besides the common TF between ADG and WBSF, we also observed common candidate genes (e.g., *LOC112449628*, *ZNF653*, *EFR3B*, *DTNB*, and *PLAG1*), which may have a role in those traits, considering that they were highlighted in the combined gene-TF network, and thus, presented more binding sites for the key TF in this study. Considering those, *PLAG1* was also found by [5], who performed a GWAS for tenderness and marbling, and [55] who associated this gene with carcass meat yield. Thus, we may suggest that these genes may be related to growth and muscle development in cattle, besides carcass meat traits.

Nevertheless, it is important to mention that this study only considered a sample of the Nelore breed animals from breeding programs in Brazil. In addition, those studied traits are well affected by the environment, which may impact DNA methylation and non-coding RNA regulation, impacting the possible candidate genes, which were not covered in this study, and the exclusion of rare variants due to the GWAS approach. In this way, further studies using a larger sample size of animals and integrative approaches, such as GWAS/Post-GWAS followed by gene expression and epigenetic marks identification, may be helpful in presenting strong candidate biomarkers for growth and meat quality traits in Nelore. 

## 5. Conclusions

This study demonstrated that ADG in Nelore cattle exhibits sufficient genetic variability to respond to selection. In contrast, due to strong environmental influences, WBSF shows limited potential for genetic improvement under conventional breeding. However, this limitation may be mitigated by the estimated genetic correlation between these traits, which, although low, suggests that selection for ADG could contribute to improving WBSF.

Identifying candidate genes, shared genomic regions, and transcription factors associated with both traits provides valuable insights into the genetic architecture of growth and meat quality in Nelore cattle and represents important targets for genomic selection. Although these findings are already significant, this study was limited to a sample of Nelore animals from breeding programs in Brazil, and future research should focus on validating these candidate genes in independent populations.

## Figures and Tables

**Table 1:** Descriptive statistics and genetic parameters for average daily gain (ADG) and Warner–Bratzler shear force (WBSF), in young Nelore steers, using a bit-characteristic animal model.

Traits	*n*	*GC*	*Mean*	*Min*	*Max*	$\sigma_{a}^{2}$	$\sigma_{p}^{2}$	$h^{2}$	*r*
ADG (kg/dia)	6077	737	0.63 ± 0.09	0.37	1.05	0.0009 ± 0.00001	0.004 ± 0.0008	0.23 ± 0.025	−0.17
WBSF (kgf)	5555	715	6.27 ± 1.96	1.60	12.84	0.28 ± 0.047	2.03 ± 0.25	0.13 ± 0.039	

***n***: number of animals; ***GC***: contemporary group; ***Min***: minimum; ***Max***: maximum; ***σ^2^_a_****:* genetic variance; ***σ^2^_p_***: phenotypic variance; ***h^2^***: heritability estimates; ***r***: genetic correlation.

**Table 2:** Genomic regions and respective candidate genes for average daily gains (ADG) explaining more than 1% of the variability in genomic breeding value in young Nelore bulls.

BTA	*n* SNP	Region (Mb)	Size (Mb)	Importance Score (%)	Gene
1	85	94.37–95.17	0.8	1.87 (1.02–2.20)	*SPATA16* (*spermatogenesis associated 16*), *ECT2* (*epithelial cell transforming 2*), *NCEH1* (*neutral cholesterol ester hydrolase 1*), *TNFSF10* (*TNF superfamily member 10*), *GHSR* (*growth hormone secretagogue receptor*), *FNDC3B* (*fibronectin type III domain containing 3B*)
2	97	103.50–104.42	0.92	2.50 (1.02–3.80)	*FN1* (*fibronectin 1*), *MIR2285L* (*microRNA mir-2285l*), *MREG* (*melanoregulin*), *LOC112443663* (*small nucleolar RNA SNORA40*), *TMEM169* (*transmembrane protein 169*), *PECR* (*peroxisomal trans-2-enoyl-CoA reductase*), *XRCC5* (*X-ray repair cross complementing 5*), *MARCHF4* (*membrane associated ring-CH-type finger 4*), *SMARCAL1* (*SWI/SNF related*, *matrix associated*, *actin dependent regulator of chromatin*, *subfamily a like 1*), *RPL37A* (*ribosomal protein L37a*)
4	109	70.52–71.56	1.04	2.15 (1.01–3.38)	*NPVF* (*Neuropeptide VF precursor*), *C4H7orf31* (*chromosome 4 C7orf31 homolog*), *CYCS* (*Cytochrome c*, *somatic*), *OSBPL3* (*Oxysterol binding protein like 3*), *LOC112446504* (*U6atac minor spliceosomal RNA*), *LOC100299757* (*EP300-interacting inhibitor of differentiation 1*), *GSDME* (*Gasdermin E*), *PALS2* (*protein associated with LIN7 2*, *MAGUK p55 family member*), *NPY* (*Neuropeptide Y*)
7	119	15.28–16.25	0.97	2.24 (1.03–3.67)	*QTRT1* (*Queuine tRNA-Ribosyltransferase Catalytic Subunit 1*), *DNM2* (*Dynamin 2*), *MIR3604-1* (*MicroRNA 3604-1*), *TMED1* (*Transmembrane P24 Trafficking Protein 1*), *C7H19orf38* (*Chromosome 7 C19orf38 Homolog*), *CARM1* (*Coactivator Associated Arginine Methyltransferase 1*), *YIPF2* (*Yip1 Domain Family Member 2*), *TIMM29* (*Translocase Of Inner Mitochondrial Membrane 29*), *SMARCA4* (*SWI/SNF Related*, *Matrix Associated*, *Actin Dependent Regulator Of Chromatin*, *Subfamily A*, *Member 4*), *LOC112447661* (*U6 spliceosomal RNA*), *LDLR* (*Low Density Lipoprotein Receptor*), *SPC24* (*SPC24 Component Of NDC80 Kinetochore Complex*), *KANK2* (*KN Motif And Ankyrin Repeat Domains 2*), *DOCK6* (*Dedicator Of Cytokinesis 6*), *ANGPTL8* (*Angiopoietin Like 8*), *RAB3D* (*RAB3D*, *Member RAS Oncogene Family*), *TMEM205* (*Transmembrane Protein 205*), *CCDC159* (*Coiled-Coil Domain Containing 159*), *PLPPR2* (*Phospholipid Phosphatase Related 2*), *SWSAP1* (*SWIM-Type Zinc Finger 7 Associated Protein 1*), *EPOR* (*Erythropoietin Receptor*), *RGL3* (*Ral Guanine Nucleotide Dissociation Stimulator Like 3*), *ODAD3* (*Outer Dynein Arm Docking Complex Subunit 3*), *PRKCSH* (*Glucosidase II Beta Subunit*), *ELAVL3* (*ELAV Like RNA-Binding Protein 3*), *ZNF653* (*Zinc Finger Protein 653*), *ECSIT* (*ECSIT Signaling Integrator*), *CNN1* (*Calponin 1*), *ELOF1* (*Transcription Elongation Factor 1*), *ACP5* (*Acid Phosphatase 5*, *Tartrate Resistant*), *LOC538435* (*heterogeneous nuclear ribonucleoproteins A2/B1*), *ZNF557* (*Zinc Finger Protein 557*), *LOC508834* (*RNA-binding protein S1*, *serine-rich domain-like*), *INSR* (*Insulin Receptor*), *ARHGEF18* (*Rho/Rac Guanine Nucleotide Exchange Factor 18*), *PEX11G* (*PEX11 Gamma*)
8	103	66.64–67.06	0.42	2.12 (1.01–2.87)	*LPL* (*lipoprotein lipase*)
11	71	73.40–74.12	0.72	2.17 (1.03–3.03)	*GAREM2* (*GRB2 associated regulator of MAPK1 subtype 2*), *RAB10* (*RAB10*, *member RAS oncogene family*), *KIF3C* (*kinesin family member 3C*), *ASXL2* (*ASXL transcriptional regulator 2*), *DTNB* (*dystrobrevin beta*), *DNMT3A* (*DNA methyltransferase 3 alpha*), *MIR1301* (*microRNA 1301*), *POMC* (*proopiomelanocortin*), *EFR3B* (*EFR3 homolog B*, *plasma membrane associated*)
14	215	22.08–23.24	1.16	2.32 (1.02–2.72)	*TCEA1* (*transcription elongation factor A1*), *LYPLA1* (*lysophospholipase 1*), *MRPL15* (*mitochondrial ribosomal protein L15*), *SOX17* (*SRY-box transcription factor 17*), *RP1* (*RP1*, *axonemal microtubule associated*), *XKR4* (*XK related 4*), *TMEM68* (*transmembrane protein 68*), *TGS1* (*trimethylguanosine synthase 1*), *LYN* (*LYN proto-oncogene*, *Src family tyrosine kinase*), *RPS20* (*ribosomal protein S20*), *LOC112449628* (*small nucleolar RNA U54*), *LOC112449630* (*U1 spliceosomal RNA*), *MOS* (*MOS proto-oncogene*, *serine/threonine kinase*), *PLAG1* (*PLAG1 zinc finger*)
19	193	47.59–48.55	0.96	1.79 (1.01–2.34)	*TANC2* (*tetratricopeptide repeat*, *ankyrin repeat and coiled-coil containing 2*), *CYB561* (*cytochrome b561*), *ACE* (*angiotensin I converting enzyme*), *ACE3* (*angiotensin I converting enzyme 3*), *KCNH6* (*potassium voltage-gated channel subfamily H member 6*), *DCAF7* (*DDB1 and CUL4 associated factor 7*), *TACO1* (*translation activator of cytochrome c oxidase I*), *MAP3K3* (*mitogen-activated protein kinase kinase kinase 3*), *LIMD2* (*LIM domain containing 2*), *STRADA* (*STE20 related adaptor alpha*), *CCDC47* (*coiled-coil domain containing 47*), *DDX42* (*DEAD-box helicase 42*), *FTSJ3* (*FtsJ RNA methyltransferase homolog 3*), *PSMC5* (*proteasome 26S subunit*, *ATPase 5*), *SMARCD2* (*SWI/SNF related*, *matrix associated*, *actin dependent regulator of chromatin*, *subfamily D*, *member 2*), *TCAM1* (*testicular cell adhesion molecule 1*), *GH1* (*growth hormone 1*), *CD79B* (*CD79b molecule*, *immunoglobulin-associated beta*), *SCN4A* (*sodium voltage-gated channel alpha subunit 4*), *LOC616254* (*intercellular adhesion molecule 2*), *LOC100140873* (*intercellular adhesion molecule 2*), *ERN1* (*endoplasmic reticulum to nucleus signaling 1*), *LOC112442810* (*small nucleolar RNA SNORD104*), *LOC112442831* (*small nucleolar RNA SNORA76*), *TEX2* (*testis expressed 2*), *PECAM1* (*platelet and endothelial cell adhesion molecule 1*)
21	88	7.37–7.85	0.48	1.96 (1.02–2.47)	*LRRC28* (*Leucine Rich Repeat Containing 28*), *TTC23* (*Tetratricopeptide Repeat Domain 23*), *SYNM* (*Synemin*, *Intermediate Filament Protein*), *IGF1R* (*Insulin Like Growth Factor 1 Receptor*)

**Table 3:** Genomic regions and respective genes for Warner–Bratzler shear force (WBSF) explaining more than 1% of the variability in genomic breeding value in young Nelore bulls.

BTA	*n* SNP	Region (Mb)	Size (Mb)	Importance Score (%)	Gene
5	36	65.08–66.99	1.91	1.50 (1.01–2.50)	*ANO4* (*Anoctamin 4*), *SLC5A8* (*Solute Carrier Family 5 Member 8*), *UTP20* (*UTP20 Small Subunit Processome Component*), *ARL1* (*ADP Ribosylation Factor Like GTPase 1*), *SPIC* (*Spi-C Transcription Factor*), *MYBPC1* (*Myosin Binding Protein C1*), *CHPT1* (*Choline Phosphotransferase 1*), *SYCP3* (*Synaptonemal Complex Protein 3*), *GNPTAB* (*N-Acetylglucosamine-1-Phosphate Transferase Subunits Alpha And Beta*), *WASHC3* (*WASH Complex Subunit 3*), *NUP37* (*Nucleoporin 37*), *PARPBP* (*PARP1 Binding Protein*), *PMCH* (*Pro-Melanin Concentrating Hormone*), *LOC112446897* (*U6 spliceosomal RNA*), *LOC112446839* (*U6 spliceosomal RNA*), *PAH* (*Phenylalanine Hydroxylase*), *ASCL1* (*Achaete-Scute Family bHLH Transcription Factor 1*), *LOC112446884* (*U1 spliceosomal RNA*)
7	3	15.77–16.38	0.61	1.18 (1.02–1.33)	*DOCK6* (*Dedicator Of Cytokinesis 6*), *RAB3D* (*RAB3D*, *Member RAS Oncogene Family*), *TMEM205* (*Transmembrane Protein 205*), *CCDC159* (*Coiled-Coil Domain Containing 159*), *PLPPR2* (*Phospholipid Phosphatase Related 2*), *SWSAP1* (*SWIM-Type Zinc Finger 7 Associated Protein 1*), *EPOR* (*Erythropoietin Receptor*), *RGL3* (*Ral Guanine Nucleotide Dissociation Stimulator Like 3*), *ODAD3* (*Outer Dynein Arm Docking Complex Subunit 3*), *PRKCSH* (*Glucosidase II Beta Subunit*), *ELAVL3* (*ELAV Like RNA-Binding Protein 3*), *ZNF653* (*Zinc Finger Protein 653*), *ECSIT* (*ECSIT Signaling Integrator*), *LOC508834* (*RNA-binding protein S1*, *serine-rich domain-like*), *INSR* (*Insulin Receptor*), *ARHGEF18* (*Rho/Rac Guanine Nucleotide Exchange Factor 18*), *PEX11G* (*PEX11 Gamma*), *ZNF358* (*Zinc Finger Protein 358*), *MCOLN1* (*Mucolipin TRP Cation Channel 1*), *PNPLA6* (*Patatin Like Phospholipase Domain Containing 6*), *CAMSAP3* (*Calmodulin Regulated Spectrin Associated Protein Family Member 3*), *XAB2* (*XPA Binding Protein 2*), *PET100* (*PET100 Cytochrome C Oxidase Chaperone*), *PCP2* (*Purkinje Cell Protein 2*)
7	61	95.68–96.41	1.22	1.84 (1.01–3.11)	PCSK1 (Proprotein Convertase Subtilisin/Kexin Type 1), CAST (Calpastatin), ERAP1 (Endoplasmic Reticulum Aminopeptidase 1), ERAP2 (Endoplasmic Reticulum Aminopeptidase 2), LNPEP (Leucyl And Cystinyl Aminopeptidase), LOC107132671 (FUN14 domain-containing protein 1-like), LIX1 (Lix1 Homolog), RIOK2 (RIO Kinase 2)
8	4	66.93–67.11	0.18	1.29 (1.18–1.58)	*LPL* (*Lipoprotein Lipase*)
11	15	73.94–74.32	0.37	1.90 (1.02–3.46)	*DTNB* (*dystrobrevin beta*), *DNMT3A* (*DNA methyltransferase 3 alpha*), *MIR1301* (*microRNA 1301*), *POMC* (*proopiomelanocortin*), *EFR3B* (*EFR3 homolog B*), *DNAJC27* (*DnaJ heat shock protein family* (*Hsp40*) *member C27*), *ADCY3* (*adenylate cyclase 3*)
14	12	23.14–23.67	0.53	1.75 (1.05–3.07)	*TMEM68* (*Transmembrane Protein 68*), *TGS1* (*Trimethylguanosine Synthase 1*), *LYN* (*LYN Proto-Oncogene*, *Src Family Tyrosine Kinase*), *RPS20* (*Ribosomal Protein S20*), *LOC112449628*, *LOC112449630*, *MOS* (*MOS Proto-Oncogene*, *Serine/Threonine Kinase*), *PLAG1* (*PLAG1 Zinc Finger*), *CHCHD7* (*Coiled-Coil-Helix-Coiled-Coil-Helix Domain Containing 7*), *SDR16C5* (*Short Chain Dehydrogenase/Reductase Family 16C Member 5*), *SDR16C6* (*Short Chain Dehydrogenase/Reductase Family 16C Member 6*), *PENK* (*Proenkephalin*)
19	2	48.99–49.38	0.39	1.31 (1.17–1.44)	*KPNA2* (*Karyopherin Subunit Alpha 2*), *C19H17orf58* (*Chromosome 19 C17orf58 Homolog*), *BPTF* (*Bromodomain PHD Finger Transcription Factor*), *PITPNC1* (*Phosphatidylinositol Transfer Protein Cytoplasmic 1*), *LOC112442758* (*5S ribosomal RNA*)
21	45	7.20–8.95	1.75	1.82 (1.03–4.08)	*LRRC28* (*Leucine Rich Repeat Containing 28*), *TTC23* (*Tetratricopeptide Repeat Domain 23*), *SYNM* (*Synemin*), *IGF1R* (*Insulin Like Growth Factor 1 Receptor*), *FAM169B* (*Family With Sequence Similarity 169 Member B*), *ARRDC4* (*Arrestin Domain Containing 4*)
23	26	2.34–3.53	1.19	1.70 (1.01–2.44)	*PRIM2* (*DNA Primase Subunit 2*), *LOC112443931*, *RAB23* (*RAB23*, *Member RAS Oncogene Family*), *BAG2* (*BAG Cochaperone 2*), *ZNF451* (*Zinc Finger Protein 451*), *BEND6* (*BEN Domain Containing 6*), *LOC112443905* (*small nucleolar RNA SNORA18*), *MIR2375* (*MicroRNA 2375*), *MIR2285J-2* (*MicroRNA 2285j-2*), *DST* (*Dystonin*)
29	27	42.24–43.76	2.52	1.67 (1.03–2.97)	*MARK2* (*Microtubule Affinity Regulating Kinase 2*), *RCOR2* (*REST Corepressor 2*), *NAA40* (*N-Alpha-Acetyltransferase 40*, *NatD Catalytic Subunit*), *COX8A* (*Cytochrome C Oxidase Subunit 8A*), *OTUB1* (*OTU Deubiquitinase*, *Ubiquitin Aldehyde Binding 1*), *MACROD1* (*Macrodomain Containing 1*), *FLRT1* (*Fibronectin Leucine Rich Transmembrane Protein 1*), *STIP1* (*Stress Induced Phosphoprotein 1*), *FERMT3* (*Fermitin Family Member 3*), *TRPT1* (*tRNA 2*′*-Phosphotransferase 1*), *NUDT22* (*Nudix Hydrolase 22*), *DNAJC4* (*DnaJ Heat Shock Protein Family* (*Hsp40*) *Member C4*), *VEGFB* (*Vascular Endothelial Growth Factor B*), *FKBP2* (*FKBP Prolyl Isomerase 2*), *PPP1R14B* (*Protein Phosphatase 1 Regulatory Inhibitor Subunit 14B*), *BAD* (*BCL2 Associated Agonist Of Cell Death*), *GPR137* (*G Protein-Coupled Receptor 137*), *KCNK4* (*Potassium Two Pore Domain Channel Subfamily K Member 4*), *ESRRA* (*Estrogen Related Receptor Alpha*), *TRMT112* (*tRNA Methyltransferase 112*), *PRDX5* (*Peroxiredoxin 5*), *CCDC88B* (*Coiled-Coil Domain Containing 88B*), *RPS6KA4* (*Ribosomal Protein S6 Kinase A4*), *SLC22A11* (*Solute Carrier Family 22 Member 11*), *SLC22A12* (*Solute Carrier Family 22 Member 12*), *NRXN2* (*Neurexin 2*), *RASGRP2* (*RAS Guanyl Releasing Protein 2*), *PYGM* (*Glycogen Phosphorylase*, *Muscle Associated*), *SPDYC* (*Speedy/RINGO Cell Cycle Regulator Family Member C*), *CAPN1* (*Calpain 1*), *MIR2407* (*MicroRNA 2407*), *SLC22A20* (*Solute Carrier Family 22 Member 20*), *POLA2* (*DNA Polymerase Alpha 2*, *Accessory Subunit*), *CDC42EP2* (*CDC42 Effector Protein 2*), *DPF2* (*Double PHD Fingers 2*), *TIGD3* (*Tigger Transposable Element Derived 3*), *SLC25A45* (*Solute Carrier Family 25 Member 45*), *FRMD8* (*FERM Domain Containing 8*), *SCYL1* (*SCY1 Like Pseudokinase 1*), *LTBP3* (*Latent Transforming Growth Factor Beta Binding Protein 3*), *ZNRD2* (*Zinc Ribbon Domain Containing 2*), *FAM89B* (*Family With Sequence Similarity 89 Member B*), *EHBP1L1* (*EH Domain Binding Protein 1 Like 1*), *KCNK7* (*Potassium Two Pore Domain Channel Subfamily K Member 7*), *MAP3K11* (*Mitogen-Activated Protein Kinase Kinase Kinase 11*), *PCNX3* (*Pecanex 3*), *SIPA1* (*Signal-Induced Proliferation-Associated 1*), *RELA* (*RELA Proto-Oncogene*, *NF-KB Subunit*)
29	21	48.85–49.71	0.86	1.38 (1.06–1.74)	*KCNQ1* (*Potassium Voltage-Gated Channel Subfamily Q Member 1*), *TRPM5* (*Transient Receptor Potential Cation Channel Subfamily M Member 5*), *TSSC4* (*Tumor Suppressing Subtransferable Candidate 4*), *CD81* (*CD81 Molecule*), *TSPAN32* (*Tetraspanin 32*), *ASCL2* (*Achaete-Scute Family bHLH Transcription Factor 2*), *TH* (*Tyrosine Hydroxylase*), *INS* (*Insulin*), *IGF2* (*Insulin Like Growth Factor 2*), *LSP1* (*Lymphocyte Specific Protein 1*), *TNNI2* (*Troponin I2*, *Fast Skeletal Type*), *CTSD* (*Cathepsin D*)

**Table 4:** Key transcription factors (TF) for average daily gain (ADG) and Warner–Bratzler shear force (WBSF) in Nelore cattle. Most representative TFs associated with genes identified for each trait, based on their biological processes and evidence in the literature.

TF	Trait	Biological Process	Literature Evidence *
*STAT3*	ADG	Eating behavior; growth hormone receptor signaling pathway	Growth hormone secretion [34]
*Egr1*	ADG and WBSF	Response to hormone stimulus; response to glucose stimulus	Differentiation of bovine skeletal muscle satellite cells [35]
*HNF4A*	WBSF	Response to glucose stimulus; glucose homeostasis; carbohydrate homeostasis	Associated with carcass quality [36]

* The literature evidence cited is only a sample of the vast available literature.

## Data Availability

None of the data were deposited in an official repository. Data may be available from the corresponding authors upon reasonable request and with prior authorization from the Alliance Nellore breeding program (www.gensys.com.br, accessed on 5 August 2025).

## Supplementary Materials

Supplementary material for this article can be found online at https://doi.org/10.5281/zenodo.16856425 (accessed on 13 August 2025).
